# Flexible wearable biosensors from poly (ionic liquid) for real-time signal monitoring

**DOI:** 10.3389/fbioe.2025.1610197

**Published:** 2025-05-21

**Authors:** Yao Liu, Siyu Han, Panpan Gu, Bai Wang, Shiyan Tian, Xiaoxu Xu, Chunmei Yang, Shujun Liu, Jianshe Hu

**Affiliations:** ^1^ Liaodong University, Dandong, China; ^2^ Shenyang Fire Science and Technology Research Institute of MEM, National Engineering Laboratory for Fire and Emergency Rescue, Shenyang, China; ^3^ Center for Molecular Science and Engineering, College of Science, Northeastern University, Shenyang, China

**Keywords:** ionic gel, ionic liquid conductive, sensing, bioelectrode, motion monitoring

## Abstract

**Introduction:**

Modern wearable electronics demand materials that are simultaneously stretchable, conductive, and environmentally robust. Hydrogels meet some of these requirements but dehydrate or freeze easily. To overcome these limitations, we prepared a poly-ionic-liquid (PIL) ionogel that integrates high elasticity with stable ionic conductivity, aiming to enable reliable, skin-compatible strain and biopotential sensing.

**Methods:**

1-Vinyl-3-butyl-imidazolium hexafluorophosphate and 1-butyl-3-methyl-imidazolium hexafluorophosphate were mixed at optimized mass ratios, followed by N,N’-methylenebis-acrylamide (cross-linker) and Irgacure-2959 (photoinitiator). The homogeneous precursor was UV-cured for 6 min to obtain a PIL ionogel (PIL-1 – PIL-4 series). Structural, thermal, mechanical, rheological, adhesive, and electrical characteristics were analysed by FT-IR, SEM, TGA/DSC, uniaxial tensile testing, rheometry, 90° peel tests, and real-time resistance measurements. Applications were evaluated by attaching the gel to human joints and by recording EMG/ECG signals.

**Results:**

The UV one-step process yielded a dense multi-cross-linked network that combined covalent and ionic interactions. The optimised sample (PIL-2) showed a fracture stress of ∼390 kPa with 320% elongation, sustaining a 500 g load without failure. It retained mass and softness after 30 days and adhered strongly (up to 90° peel strength >4 N) to glass, metals, and skin—even underwater. Electrical tests gave a gauge factor of 1.94 (0–100%), 3.98 (100–200%), and 4.04 (200–320%), with 400 ms response and 500 ms recovery. The gel monitored finger (30°/90°), wrist, and elbow motions reproducibly, functioned as a bioelectrode capturing stable EMG/ECG with clear PQRST waves, and reliably transmitted Morse code via hand gestures.

**Discussion:**

The solvent-free PIL ionogel couples mechanical toughness, wide-range elasticity, and stable ionic pathways, outperforming water-rich hydrogels in thermal/long-term stability. Its strong, humidity-tolerant adhesion eliminates extra fixatives, while rapid, high-gain strain transduction and low-impedance skin contact enable multimodal biosensing. These attributes position the material for next-generation flexible electronics, real-time health monitoring, and gesture-based human-machine interfaces.

## 1 Introduction

At the forefront of contemporary technological advancements, flexible mechanical strain sensors, as an innovative technology emerging at the intersection of materials science and electronic engineering, are gaining significant attention (Ma et al., 2023; Wang et al., 2023; Xue et al., 2023; Chang et al., 2024; Tang et al., 2021). These sensors can efficiently convert external mechanical changes into electrical signals, showcasing immense potential in applications such as wearable devices, medical monitoring, and intelligent robotics. Conventional sensor fabrication approaches typically involve embedding nanoscale conductive fillers, such as carbon nanotubes, silver wires, or graphene, into elastic matrices to create composite materials capable of accurately detecting subtle deformations. While this approach is theoretically sound, practical challenges arise due to incompatibility between materials, leading to structural fragility, limited stretchability, and optical opacity of the fillers. These issues severely hinder their practical implementation in critical applications (He et al., 2024; Li et al., 2019; An et al., 2025; de Oliveira et al., 2020; Zhang et al., 2018).

In contrast, ionic conductive sensors demonstrate enhanced stretchability and optical transparency. Unlike traditional electronic conductors, ionic conductors rely on ion mobility for electrical conduction. Common examples include metal salt solutions and ionic liquids, while ionic conductive hydrogels have gained significant attention due to their flexibility, transparency, and biocompatibility (Wang D. et al., 2024; Peng et al., 2022; Yuan et al., 2024; Jin et al., 2024; Gao et al., 2024). For instance, Kang et al. developed a novel, flexible, and highly sensitive pressure sensor. This sensor utilizes an elastic dielectric layer, which exhibits an exceptionally high dielectric constant and uniformity due to the incorporation of a synthesized ionic liquid-grafted silicone oil (denoted as LMS-EIL) (Kang et al., 2021). However, hydrogels are susceptible to freezing at low temperatures and dehydration at high temperatures, which diminishes their stretchability and conductivity, thereby limiting their potential in flexible strain sensor applications (Zhou et al., 2022; Satani et al., 2020; Roata et al., 2018).

Ionic liquids, renowned for their negligible vapor pressure, exceptional thermal stability, and expansive electrochemical windows, present unparalleled advantages in creating ionogels that not only exhibit enhanced environmental adaptability but also significantly improve overall performance. When integrated with cross-linked polymers, these ionic liquids address the performance limitations often encountered with traditional hydrogel sensors, such as high water content and susceptibility to evaporation (Ichikawa et al., 2019; Li et al., 2024; Alyami et al., 2023; Chen et al., 2024; Kang et al., 2019; Teng et al., 2019). The unique physicochemical properties of ionic liquids not only enhance the ionic conductivity of the gels but also contribute to increased mechanical robustness and thermal durability, making them suitable for a diverse range of applications. Moreover, the tunable nature of ionic liquids allows for precise adjustments in their properties, enabling the development of customized ionogels tailored for specific functionalities.

Ionic conductive gels are typically synthesized by incorporating ionic liquids into elastomers, which can significantly modulate their properties. For instance, Guo et al. developed an innovative ionogel by embedding halloysite nanotubes (HNT) into a cellulose-based ionic liquid matrix. Here, HNT acts as a conductive enhancer, facilitating an oriented arrangement within the ionic liquid that induces anisotropy. This anisotropic configuration not only improves the mechanical properties but also enhances the conductivity and thermal stability of the ionogel (Huang et al., 2019). While these methods show great promise for optimizing material properties, significant challenges remain, including the complexity of polymer synthesis and the need for precise process control. Overcoming these challenges is critical for harnessing the full potential of ionogels in advanced applications such as flexible electronics and sensing technologies, where performance reliability and adaptability in diverse environments are essential (Cui et al., 2024; Qin et al., 2024; Wu et al., 2024; Zhang et al., 2025; Zhao et al., 2024; Wang Z. et al., 2024).

This study focuses on the preparation of ionic conductive gels through *in-situ* photopolymerization using imidazolium-based ionic liquids, 1-vinyl-3-butylimidazolium hexafluorophosphate and 1-butyl-3-methylimidazolium hexafluorophosphate, and investigates their synthesis and performance. The preparation process is streamlined, requiring only a single step of UV light polymerization, which simplifies traditional synthesis methods. The resulting ionogels exhibit several key properties, including self-adhesive stretchability, strong antibacterial activity, and effective stress-strain sensing capabilities, meeting the demanding requirements for flexible strain sensor materials. In sensor applications, the ionogel demonstrates good electromechanical performance, such as high sensitivity, low hysteresis, and repeatability, allowing for accurate monitoring of human joint movements (e.g., fingers, wrists, and elbows). Furthermore, the ionogel has been adapted into bioelectrodes for bioelectrical signal acquisition. In conclusion, the proposed method for preparing ionic conductive gels is both simple and efficient, offering significant potential for applications in flexible electronics, biosensors, and smart materials, and providing a novel technological foundation for these fields.

## 2 Materials and methods

### 2.1 Materials

1-Vinyl-3-butylimidazolium hexafluorophosphate ([VBIm][PF_6_], 98%), 1-butyl-3-methylimidazolium hexafluorophosphate ([BMIm][PF_6_], 97%), N,N′-methylenebisacrylamide (MBA, 97%), and 2-hydroxy-4′-(2-hydroxyethoxy)-2-methylpropiophenone (photoinitiator, Irgacure-2959, 98%) were purchased from Aladdin Reagent Co., Ltd. (Shanghai, China) and Sinopharm Chemical Reagent Co., Ltd. (China).

### 2.2 The synthesis of PIL ionic gel

The synthesis of the ionic liquid gel was initiated by preparing ionic liquid monomers, specifically 1-vinyl-3-butylimidazolium hexafluorophosphate ([VBIm][PF_6_]) and 1-butyl-3-methylimidazolium hexafluorophosphate ([BMIm][PF_6_]), in small sample vials at predetermined mass ratios. The mixture was subjected to continuous heating under vigorous stirring until a homogeneous, transparent solution was achieved, resulting in the formation of the precursor liquid. Following this, photoinitiator (Irgacure-2959) and the crosslinking agent N,N′-methylenebisacrylamide (MBA) were introduced into the precursor solution. The resultant clear solution was promptly transferred into pre-designed molds and irradiated under ultraviolet light for a duration of 6 min to facilitate the polymerization process, ultimately yielding the final ionic liquid gel. Detailed information regarding the specific mass ratios and corresponding sample nomenclature is provided Table 1.

**TABLE 1:** Feed ratio of ionogel.

Sample	[VBIm][PF_6_] (g)	[BMIm][PF_6_] (g)	Irgacure-2959 (Wt%)	MBA (Wt%)
PIL-1	1.60	2.00	0.10	0.10
PIL-2	1.60	2.00	0.10	0.20
PIL-3	1.60	2.00	0.15	0.10
PIL-4	1.60	2.00	0.15	0.15

### 2.3 General characterizations

The structural and thermal properties of the samples were systematically characterized using a suite of advanced analytical techniques. Fourier transform infrared (FTIR) spectra were acquired in the spectral range of 4,000–500 cm^−1^ employing a Spectrum One spectrometer (PerkinElmer, United States). Morphological analysis was conducted via scanning electron microscopy (SEM) using a HITCHI SU 8010 system. Thermogravimetric analysis (TGA) was performed on a Netzsch 209C instrument under a nitrogen atmosphere, with a temperature gradient spanning from 50°C to 800°C. The thermal behavior of the ionic liquid gel was further investigated through differential scanning calorimetry (DSC) using a Netzsch DSC-204 analyzer, with measurements conducted under a continuous nitrogen flow. The temperature was varied from −90°C to 60°C at a controlled heating rate of 10 K·min^−1^. To evaluate the stability of the ionogels, the mass of freshly synthesized samples was meticulously recorded. Subsequently, the gels were transferred into glass Petri dishes and maintained under ambient conditions for a period of 30 days.

### 2.4 The mechanical properties test

The mechanical behavior of the materials under uniaxial loading conditions was evaluated utilizing an electronic universal testing machine (WDW-02), with experimental protocols rigorously aligned to the Type I specimen specifications prescribed by national standards. A consistent tensile rate of 10 mm·min^−1^ was maintained throughout the testing process to guarantee the accuracy and repeatability of the obtained results. Specimens were fabricated in the standardized dumbbell geometry to facilitate the measurements.

### 2.5 The rheological properties test

A comprehensive analysis of the mechanical characteristics of poly(ionic liquid) ionogels was conducted employing an Anton Paar MCR302 rheometer. The viscoelastic response of the elastomer was initially assessed through the determination of the storage modulus (G′) and loss modulus (G″) across a frequency spectrum spanning from 0.1 rad/s to 100 rad/s, maintaining a constant shear strain of 0.5%. Following this, the self-healing properties of the material were investigated by subjecting it to oscillatory strain amplitudes varying from 0.01% to 1,000% at a fixed angular frequency of 10 rad/s. The restoration of mechanical integrity post-deformation was systematically evaluated by observing the variations in G′ and G''.

### 2.6 The adhesion test

The adhesive behavior of the gel material was systematically explored through the selection of diverse substrates, encompassing glass, rubber, plastic, copper, and iron. Ionogel specimens, measuring 5 cm × 2 cm with a uniform thickness of 3 mm, were subjected to a 90° peel test utilizing a universal testing apparatus (WDW-02) to evaluate their interfacial bonding characteristics.

### 2.7 The sensing testing

Real-time monitoring of resistance changes in all strain sensors was conducted employing a CHI760E digital electrochemical workstation, which operated at an output voltage of 1 V. The sensitivity of the stretchable strain sensor fabricated from poly(ionic liquid) (PIL) ionogel was evaluated through the application of the standard gauge factor (GF) equation: GF = (R - R_0_)/(R_0_ × ε). In this formulation, R_0_ (Ω) signifies the baseline resistance of the PIL elastomer before deformation, R (Ω) indicates the resistance measured during strain application, and ε represents the magnitude of the applied strain.

## 3 Results and discussion

### 3.1 The structural and thermal properties

**FIGURE 1:**
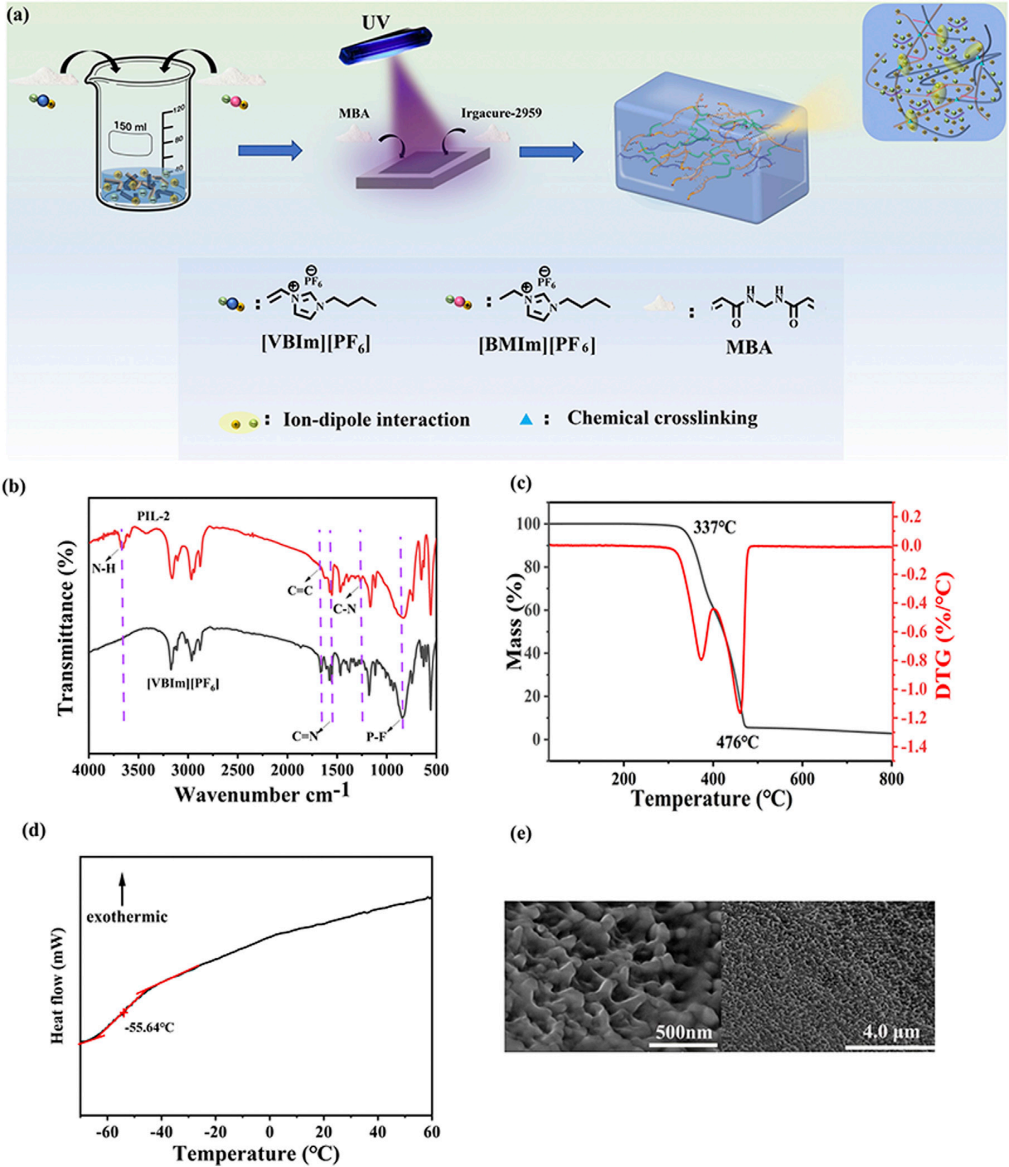
Structural and thermal characterization of the ionogels. **(a)** Synthesis Scheme, **(b)** FT-IR spectra of PIL-2 , **(c)** TGA curves of PIL-2, **(d)** DSC curve of PIL-2, and **(e)** SEM imagines of PIL-2 i gel.

The synthesis of the ionogel was based on the rigorous selection and optimization of ionic liquid monomers, crosslinker, and photoinitiator. The specific procedure included the following steps: First, 1-vinyl-3-butylimidazolium hexafluorophosphate and 1-butyl-3-methylimidazolium hexafluorophosphate were mixed in a specific ratio and subjected to heating. Subsequently, a crosslinker (MBA) and a photoinitiator (Irgacure-2959) were added to the mixture, and the curing reaction was completed through ultraviolet (UV) light irradiation, yielding the target ionogel. In the crosslinked structure of the gel, the chemical crosslinking effect of MBA synergized with the ionic interactions of the ionic liquids, significantly enhancing the functional properties and durability of the material. This synergistic effect enabled the ionogel to exhibit outstanding mechanical performance and stability in various application scenarios (Figure 1A). To characterize the internal chemical structure of the material and confirm the polymerization of [VBIm][PF_6_], The peak near 1,665 cm^−1^ is attributed to the stretching vibration of C=N, the peak around 1,261 cm^−1^ corresponds to the stretching vibration of C-N, the peak near 3,661 cm^−1^ corresponds to the bending vibration of N-H, the peak around 840 cm^−1^ is due to the stretching vibration of P-F, and the peak near 1,671 cm^−1^ arises from the stretching vibration of C=C. After UV irradiation, the peak at 1,671 cm^−1^ disappears due to the cleavage and polymerization of C=C. This analysis indicates that the molecular structure of the PIL gel meets our expectations. (Figure 1B).

Ionic liquid gels are good ion-conducting materials, and their superior thermal stability enables their application in harsh environments. To evaluate the thermal properties, thermogravimetric analysis (TGA) was conducted on PIL-2. The results indicated that the decomposition temperature of the ionic liquid gel began at 337°C, demonstrating its high thermal stability and broad operational range, making it suitable for use in various extreme conditions. Furthermore, the decomposition rate exhibited two distinct peaks. The first peak was attributed to the loss of liquid ions embedded within the gel network as the temperature increased, while the second peak corresponded to the breakdown of the solid three-dimensional network, leading to the complete decomposition of the remaining ionic liquid and the solid network structure (Figure 1C).

The glass transition temperature (*T*_g_) is the temperature at which a material transitions from a glassy state to a rubbery state. The *T*_g_ is an intrinsic property of amorphous polymers and represents the macroscopic manifestation of changes in molecular motion within the material. When the temperature is below *T*_g_, the molecular chains and segments within the material are immobilized, while atoms or groups within the molecules can vibrate around their equilibrium positions. This state is referred to as the glassy state, where the amorphous regions of the material are frozen. In the glassy state, the material exhibits no viscosity or elasticity and behaves as a rigid solid, similar to glass. As the temperature increases and reaches *T*_g_, the segments within the material begin to move, although the molecular chains remain immobile. This state is known as the rubbery state, where the material’s internal structure becomes unfrozen. In the rubbery state, the material exhibits high elasticity, with significant deformation occurring, and remains relatively stable over a certain temperature range. The DSC curve of PIL-2 gel was measured over the temperature range of −90°C to 60°C. The *T*_g_ of the gel was determined to be −55.64°C, indicating that the material remains in the rubbery state at room temperature. This property endows the material with high elasticity and a broad operational range (Figure 1D). To investigate the internal microstructure of the ionic liquid gel, the dried PIL-2 gel was examined using scanning electron microscopy (SEM). The results revealed that the gel exhibited a porous three-dimensional network structure. This porous architecture facilitates ion transport when the gel is employed as a flexible sensor, thereby enhancing its sensitivity (Figure 1E).

### 3.2 The mechanical properties

**FIGURE 2:**
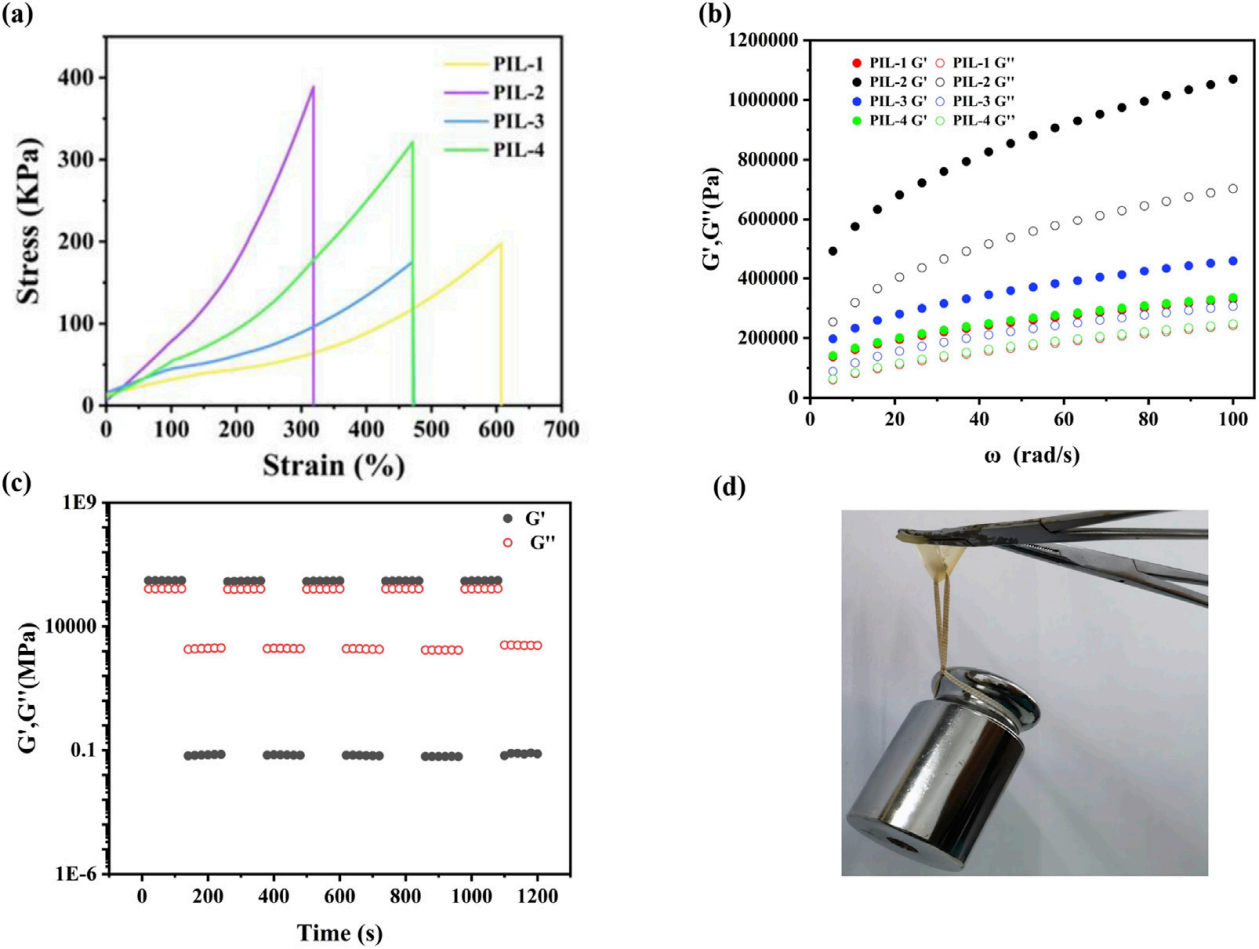
Mechanical properties of the ionogels **(a)** Stress-strain curves of ionogels, **(b)** Comparison of toughness and Young's modulus of ionogels, **(c)** Cyclic tensile tests at 100% strain without any resting time between cycles of PIL-2, **(d)** Load-bearing diagram of PIL-2.

The tensile stress-strain curves of the materials revealed that as the elongation at break increased, the fracture stress decreased, indicating poorer mechanical performance. PIL-1 exhibited an elongation at break of 610%, but its fracture stress was only 198 kPa. In contrast, PIL-2 showed the lowest elongation at break of 320%, but its mechanical properties were significantly enhanced, with a fracture stress of 390 kPa. Under the condition of the same photoinitiator, a higher crosslinker content resulted in a denser crosslinked network, leading to increased fracture stress and reduced elongation at break in the ionic liquid gels. This trend was also observed in PIL-3 and PIL-4, where the increase in crosslinker content notably improved the fracture stress. Additionally, a comparison between PIL-1 and PIL-3 demonstrated that an increase in photoinitiator content was detrimental to the enhancement of fracture stress. The denser the ionogel network, the stronger its structural stability. For sensing materials, in addition to an appropriate elongation at break, a stable structure is essential to ensure consistent and reliable sensing performance (Figure 2A).

To explore the influence of reaction conditions on the PIL ionogel system, frequency sweep tests were conducted to examine viscoelastic parameters, particularly the storage modulus (G′) and loss modulus (G″). Measurements were performed over an angular frequency range of 0.1–100 rad/s. The results indicated that G′ increased with frequency, suggesting the presence of a stable three-dimensional crosslinked network within the ionogels. Furthermore, G′ consistently exceeded G″, confirming the elastic behavior of the ionogels across all doping ratios. A comparison between PIL-1 and PIL-2 revealed that PIL-2 exhibited a higher G′ than PIL-1. This improvement was attributed to the increased crosslinker content, which, despite the use of the same photoinitiator, resulted in a denser crosslinked network and enhanced rigidity of the ionogel (Figure 2B). Similarly, PIL-3 demonstrated a higher G′ compared to PIL-4, further highlighting the significant role of crosslinker concentration in improving the mechanical properties. However, the comparison between PIL-1 and PIL-3 indicated that an increase in photoinitiator content had a detrimental effect on the mechanical performance of the material.

When subjected to sustained constant shear stress, the gels displayed thixotropic properties, characterized by a gradual reduction in viscosity over time, followed by recovery upon stress removal. Cyclic strain time sweep analyses demonstrated that the G′ and G″ of the hydrogels declined markedly under high strain but swiftly rebounded under low strain. This behavior suggested a shift from an elastic to a viscous state under high strain, coupled with pronounced self-repair capabilities under low strain. These observations underscored the dynamic and reversible structural adaptations of the hydrogels under varying strain levels. For example, rheological evaluations of PIL-2 revealed that its structural integrity remained intact after repeated loading cycles, attributed to its dense crosslinked network (Figure 2C). Moreover, PIL-2 ionogel showed superior mechanical strength, with the ability to sustain a 500 g load (Figure 2D).

### 3.3 Adhesion and the stability

**FIGURE 3:**
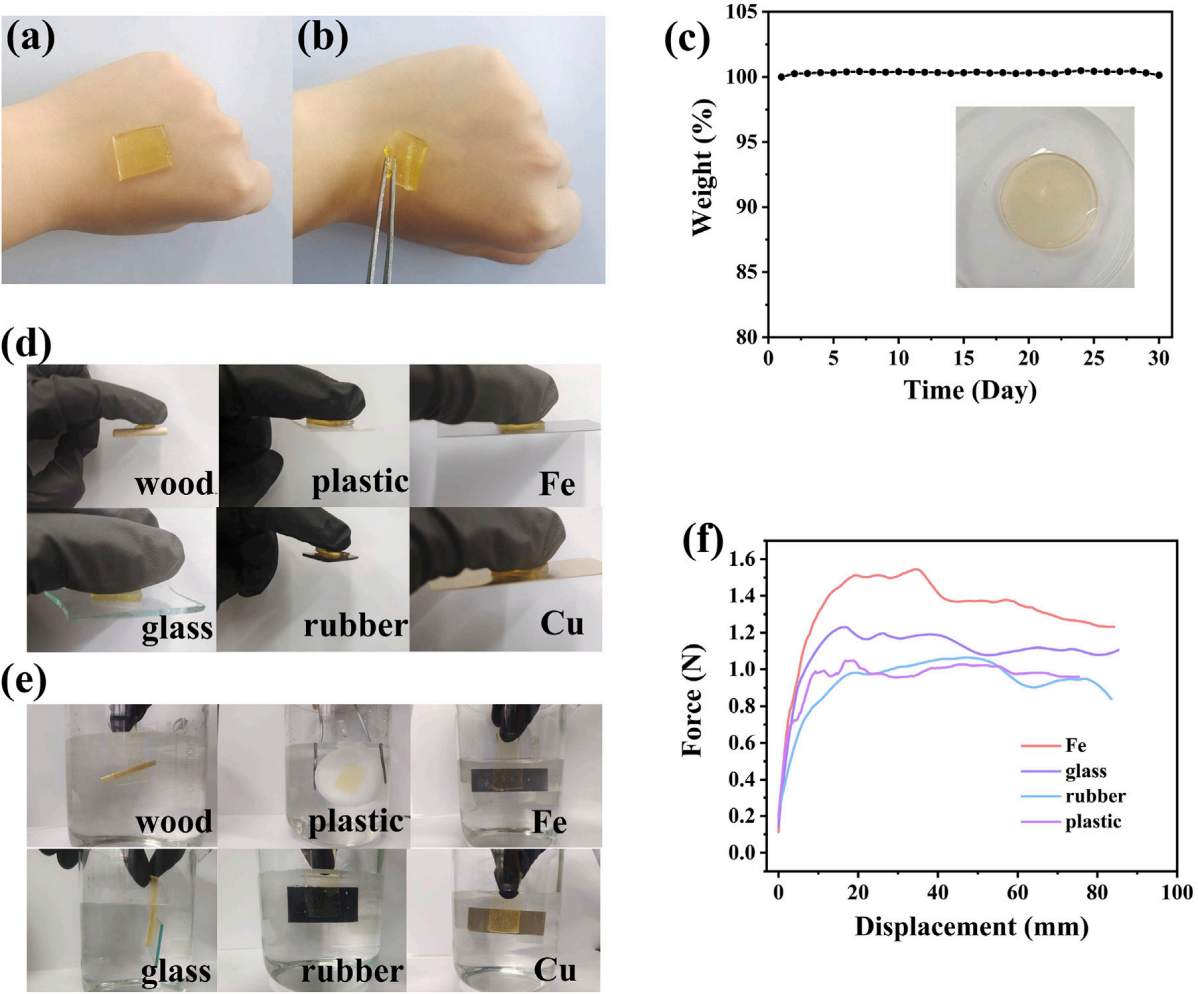
The adhesion and stability properties of PIL-2. **(a,b)** skin sensitivity test results **(c)** room temperature stability test result, **(d)** Adhesive performance on different materials, **(e)** adhesive performance underwater, **(f)** peel test results.

The ionogel sensing material exhibited good adhesion to the skin, and no irritation or damage was observed even after 30 min of application (Figures 3A,B). A 30-day stability test was conducted on the PIL-2 material. The mass of the material remained largely unchanged after 30 days at room temperature, with no significant water absorption or solvent evaporation observed. Additionally, its macroscopic morphology and softness showed no notable alterations, indicating that the material maintained long-term stability at room temperature. Unlike hydrogels, whose properties change due to solvent evaporation, the ionogel retained its characteristics over time (Figure 3C).

The prepared PIL-2 ionogel demonstrated the ability to adhere easily and firmly to various substrates, including skin, rubber, wood, copper (Cu), iron (Fe), glass, and plastics, indicating its good adhesive performance (Figure 3D). The adhesive properties were likely attributed to intermolecular interactions between the functional groups of the ionogel and different groups on the substrate surfaces, such as van der Waals forces, hydrogen bonding, electrostatic interactions, cation-π interactions, ion-dipole interactions, and dipole-dipole interactions. The good flexibility and extensibility of the ionogel enabled it to effectively embed into the uneven microporous surfaces of various substrates, forming strong connections and adhesion. Due to the complex physical and chemical mechanisms, the prepared ionogel exhibited adhesion to a wide range of materials.

For flexible sensing materials, adhesive properties are essential. However, adhesion strength is often influenced by environmental humidity, making it crucial for the ionogel to maintain adhesion under high humidity or in water. The ionogel retained good adhesive performance even underwater. The presence of abundant polar groups in the ionogel facilitated strong adhesion to different substrates through various interactions, including hydrophobic interactions (Figure 3E). A 90° peel test was employed to quantitatively characterize the adhesion strength of the ionogel to various materials, including glass, plastic, rubber, and iron. The adhesive force during peeling was used to evaluate the interfacial bonding strength. The synergistic effects of chemical bonds and energy dissipation during the peeling process explained the strong adhesion of the ionogel to different substrates. The ionogel adhered to iron exhibited the highest adhesion strength (Figure 3F).

### 3.4 Electrical and sensing properties

**FIGURE 4:**
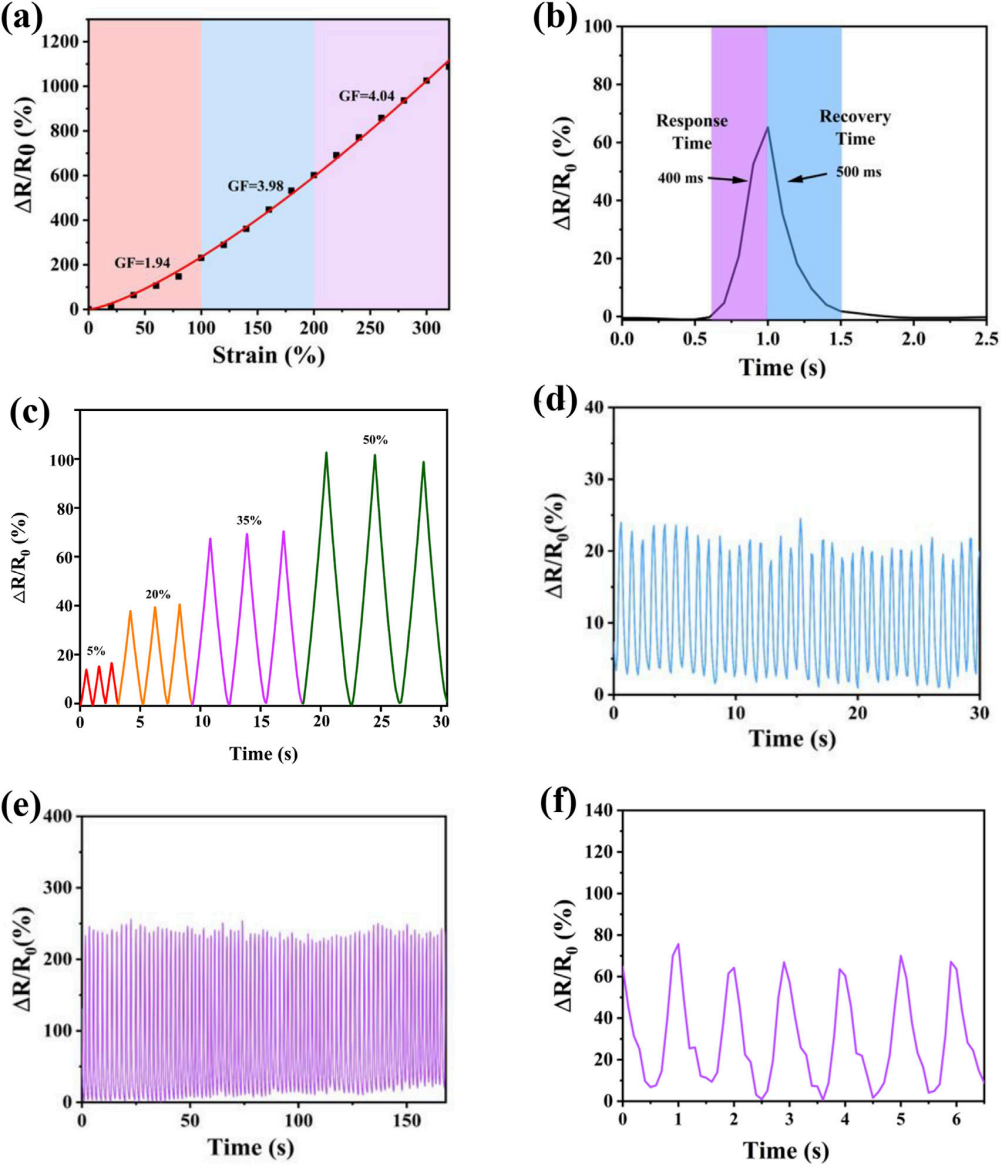
**(a)** variations in relative resistance correlated with different strain levels, **(b)** the response and recover behavior in 1 cycle of tensile, **(c)** multi-cycle tests of relative resistance variation, strain sensor upon stretching to different maximum strains: maximum strain of 25 %, 50 %, 75 %, 100%, **(d)** Results of Cyclic Compression, **(e)** cyclic tensile test results **(f)** underwater tensile test results.

High sensitivity is a prerequisite for flexible sensors based on ionogels. The relative change in resistance (ΔR/R_0_) exhibited a good linear relationship with the applied strain in segmented regions. The gauge factor (GF), calculated as the ratio of ΔR/R0 to tensile strain, served as a key parameter to quantify sensitivity. For PIL-2, the GF value was 1.94 in the strain range of 0%–100%, 3.98 in the range of 100%–200%, and 4.04 in the range of 200%–320%. These values represented varying sensitivities under different strain levels. At lower strains (0%–100%), the changes induced by the stretching of ions in the ionic liquid were minimal, resulting in a smaller GF value. At higher strains (200%–320%), the separation of anions and cations in the ionic liquid likely disrupted the conductive pathways, leading to an increase in relative resistance and a higher GF value. The high sensitivity of the ionogel material enabled its potential use in a wider range of applications (Figure 4A). The response time of PIL-2 was evaluated by measuring the relative change in resistance. The material exhibited a response time of 400 ms and a relaxation time of 500 ms at a 65% relative resistance change. These results indicated that the material could rapidly respond to large deformations within a short time frame (Figure 4B).

The conductive behavior of the PIL ionogel was attributed to the unrestricted mobility of its internal ions, which established uninterrupted conductive networks throughout the gel structure. Among its key attributes as a flexible conductive material was its ability to detect mechanical strain. To assess this property, the relative resistance variation (ΔR/R0) was measured across a range of strain levels. A proportional rise in the sensing signal amplitude was observed as the applied tensile strain escalated from 5% to 50%, highlighting the material’s remarkable sensitivity and consistent performance under repeated testing (Figure 4C).

PIL-2 demonstrated the ability to undergo 80 cycles of stretching and compression within 3 min while maintaining its performance with minimal degradation. This exceptional stability was closely linked to its structural and mechanical properties. Among the tested groups, PIL-2 contained the highest proportion of crosslinking agents, resulting in a denser network structure that effectively preserved its original morphology. This structural integrity enabled rapid and efficient recovery to its initial state after deformation. Although the increased crosslinking density slightly reduced its maximum strain, PIL-2 still exhibited an impressive elongation at break of 320%, which proved fully sufficient for practical applications in motion sensing (Figures 4D,E). During molecular design, long-chain fluorinated ionic liquids were selected to enhance the material’s underwater stability and performance. PIL-2 was subjected to underwater sensing tests, where it demonstrated consistent and stable sensing capabilities. The material maintained reliable sensing performance both in air and underwater, significantly broadening its potential applications. This adaptability allows it to meet diverse user requirements and accommodate various sensor-wearing scenarios (Figure 4F).

### 3.5 Motion sensing

**FIGURE 5:**
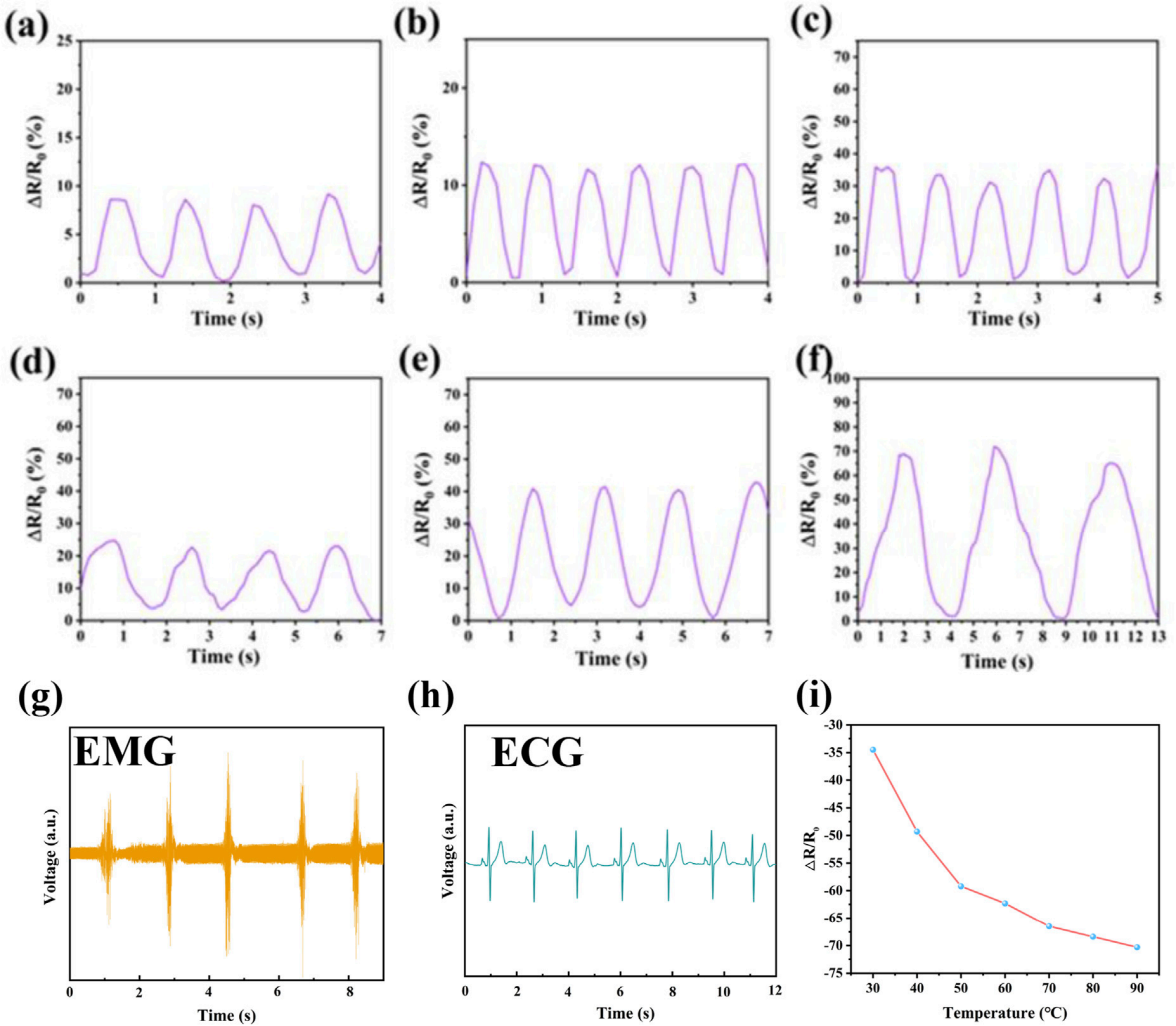
**(a)** Finger bend of 30°, **(b)** finger bend of 90°, **(c)** wrist lift, **(d)** wrist press down, **(e)** elbow bend of 90°, **(f)** elbow maximum bend, **(a,b,g,h)** results of the PIL ionic gel as bioelectrodes for signal acquisition. **(i)** relative resistance changes of PIL ionic gel at different temperatures.

Flexible sensors with good overall performance are increasingly aligned with developmental trends and demands, particularly for monitoring human joint movements and subtle physiological activities. To explore broader application prospects, we conducted motion sensing tests on PIL-2, focusing on its performance under stretching and pressing conditions. When the finger was bent sequentially at 30° and 90° (Figures 5A,B), the sensor exhibited a corresponding increase in relative resistance change, with the electrical signal demonstrating high repeatability and stability. Upon straightening the finger, the relative resistance returned completely to its initial value. Similarly, when the sensor was attached to the wrist (Figures 5C,D) or elbow (Figures 5E,F), it accurately detected the bending amplitudes of these joints. These results confirm that PIL-2 is capable of highly specific and reliable sensing. PIL ionogel also serves as a bioelectrode for detecting physiological electrical signals from the human body, which is critical for health monitoring. Using PIL ionogel as a biocompatible electrode (Figures 5G,H), we conducted electromyography (EMG) and electrocardiography (ECG) signal detection on healthy male volunteers. The results showed that the acquired ECG and EMG signals were stable and reproducible, with clear PQRST waveforms. Notably, the PIL ionogel’s ability to effectively prevent dehydration makes it an ideal material for skin electrodes. This property enhances its suitability for physiological signal detection, offering promising prospects for monitoring and assessing physical conditions. Additionally, the relative change in resistance of the PIL ionogel with temperature indicated a decrease in resistance as temperature increased, demonstrating its good temperature responsiveness (Figure 5I).

**FIGURE 6:**
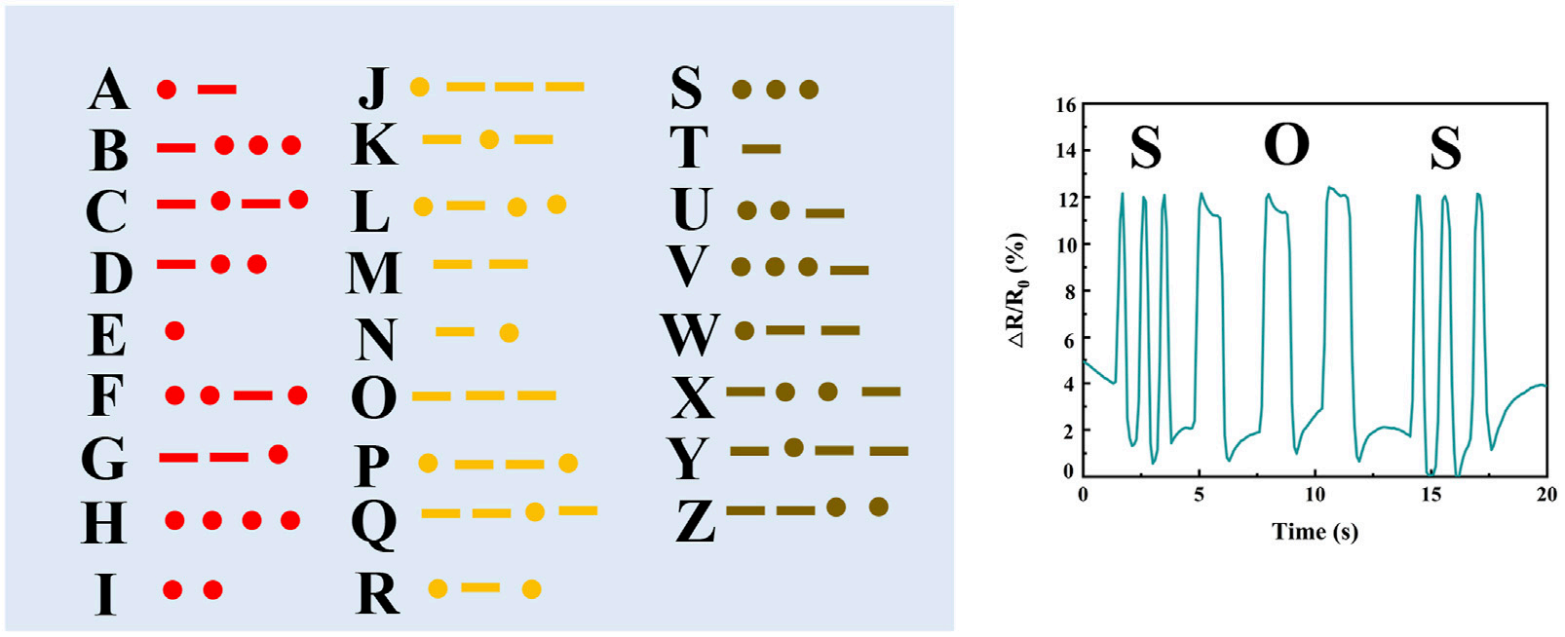
Results of Morse code transmission.

Furthermore, the PIL ionogel was successfully applied in transmitting Morse code, showcasing its ability to deliver stable and accurate signal transmission (Figure 6).

## 4 Conclusion

In summary, this study designed and synthesized a novel poly ionic liquid (PIL)-based ionogel that seamlessly integrates flexibility and functionality. By optimizing the ratios of ionic liquid monomers, crosslinkers, and photoinitiators, the material achieved a robust crosslinked network through a green and efficient one-step UV crosslinking process. The combination of chemical crosslinking effects and ionic interactions significantly enhanced the mechanical properties and durability of the ionogel. The multi-crosslinked network structure ensured good mechanical strength, while the uniform distribution of ionic liquids greatly improved its conductivity. The ionogel demonstrated exceptional strain sensing capabilities (GF = 4.04) and temperature responsiveness, enabling precise environmental monitoring. Additionally, as a biopotential electrode patch, it efficiently captured electromyography (EMG) and electrocardiography (ECG) signals, providing accurate data support for health monitoring. Furthermore, the material’s ability to stably and accurately transmit Morse code through gestures expanded its application scope. This PIL-based ionogel emerges as a promising candidate for flexible electronics, health monitoring, and human-machine interaction applications, paving the way for innovative advancements in these fields.

## Data Availability

The raw data supporting the conclusions of this article will be made available by the authors, without undue reservation.
